# Assessment of NMDA receptor genes (*GRIN2A*, *GRIN2B* and *GRIN2C*) as candidate genes in the development of degenerative lumbar scoliosis

**DOI:** 10.3892/etm.2013.910

**Published:** 2013-01-21

**Authors:** KI-TACK KIM, JINSUNG KIM, YOO JIN HAN, JUN HO KIM, JONG SEOK LEE, JOO-HO CHUNG

**Affiliations:** 1 Department of Orthopedic Surgery, Spine Center, School of Medicine, Kyung Hee University Hospital at Gangdong, Seoul 134-727;; 2 Department of Physical Medicine and Rehabilitation; School of Medicine, Kyung Hee University, Seoul 130-701, Republic of Korea; 3 Department of Pharmacology and Kohwang Medical Research Institute; School of Medicine, Kyung Hee University, Seoul 130-701, Republic of Korea; 4 Department of Emergency Medicine, School of Medicine, Kyung Hee University, Seoul 130-701, Republic of Korea

**Keywords:** *GRIN2*, single nucleotide polymorphism, degenerative lumbar scoliosis, Korean

## Abstract

Degenerative lumbar scoliosis (DLS) progresses with aging after 50–60 years. The genetic association of DLS remains largely unclear. In this study, the genetic association between glutamate receptor, ionotropic, *N*-methyl D-aspartate (*NMDA*, *GRIN*) receptor genes and DLS was investigated. A total of 9 coding single nucleotide polymorphisms (cSNPs) in NMDA receptor genes [*GRIN2A* (rs8049651, Leu425Leu; rs9806806, Tyr730Tyr); *GRIN2B* (rs7301328, Pro122Pro; rs35025065, Asp447Asp; rs1805522, Ile602Ile; rs1806201, Thr888Thr; rs1805247, His1399His); and *GRIN2C* (rs689730, Ala33Ala; rs3744215, Arg1209Ser)] were selected and genotyped using direct sequencing in 70 patients with DLS and 141 healthy controls. Multiple logistic models (codominant, dominant and recessive) were calculated for the odds ratio (OR), 95% confidence interval (CI) and corresponding P-values. The SNPStats, SNPAnalyzer and HelixTree programs were used for the evaluation of the genetic data. Among the SNPs examined, no significant associations were observed between the *NMDA* receptor genes and DLS. When the patients were divided into two groups according to clinical characteristics based on Cobb’s angle (<20° or ≥20°) and lateral listhesis (<6 mm or ≥6 mm), associations were observed between rs689730 of *GRIN2C* and Cobb’s angle (codominant, P=0.038; dominant, P=0.022) and between rs7301328 of *GRIN2B* and lateral listhesis (codominant, P=0.003; dominant, P=0.015; recessive, P=0.015). These results indicate that the *GRIN2A*, *GRIN2B* and *GRIN2C* genes do not affect the development of DLS. However, the *GRIN2C* gene may be associated with Cobb’s angle, while the *GRIN2B* gene may be associated with lateral listhesis.

## Introduction

Degenerative scoliosis develops after skeletal maturity without a previous history of scoliosis and typically presents in the lumbar or thoracolumbar spine (1,2). The etiology of degenerative lumbar scoliosis (DLS) remains unclear, although several factors, including hormones, genetics and degenerative diseases of the spine, may be involved (3). It has been suggested that the cause of DLS is associated with disc degeneration and osteoporosis (4).

During an individual’s lifetime, modeling, remodeling and repair of the bone occur as a result of the activity of osteoclasts and osteoblasts (5). The control of bone remodeling is performed by a few neurotransmitters, such as glutamate, via corresponding receptors in the bone cells (6). Glutamate is a major excitatory neurotransmitter in the central nervous system (CNS) (7) and acts through a variety of receptors including N-methyl-D-aspartate (NMDA) receptors. The NMDA receptors (NMDARs) are composed of NMDAR1, NMDAR2 and NMDAR3 and are expressed in the bone cells (8,9). In addition, glutamate is a known regulator of the maturation and differentiation of osteoclasts and osteoblasts (10,11).

We hypothesized that genetic polymorphisms in the *NMDAR* genes may be associated with DLS. However, there have been no published studies concerning the association between DLS and single nucleotide polymorphisms (SNPs) of *NMDAR* genes. Therefore, the 9 coding SNPs (cSNPs) of *NMDAR2A* (*GRIN2A*), *NMDAR2B* (*GRIN2B*) and *NMDAR2C* (*GRIN2C*) were analyzed in 70 patients with DLS and 141 control subjects.

## Materials and methods

### Subjects

The DLS group comprised 70 unrelated individuals (10 males and 60 females), aged 52–82 years (mean ± SD, 67.63±7.05 years). The DLS patients were selected from the Spine Center of Kyung Hee University East-West Neo Medical Center (Seoul, Korea) and the National Medical Center (Seoul, Korea), out of ∼9,000 patients per year over three years. Each patient was diagnosed by two specialized spine surgeons and all patients fulfilled the physical examination and radiographic criteria (Cobb’s angle, >10°). Informed consent was obtained from all individuals according to the Declaration of Helsinki guidelines. The study was approved by the Ethics Review Committee of the Medical Research Institute, Kyung Hee University Medical Center (Seoul, Korea). The control group comprised 141 individuals (25 males and 116 females), aged 53–85 years (mean ± SD, 66.74±8.23 years). Control group individuals were recruited following a general health check-up program to confirm that they had no clinical evidence of DLS according to radiographic criteria. The ages and genders of the control group individuals were matched to those of the DLS group (Table I).

### SNP genotyping

The cSNPs of *GRIN2A*, *GRIN2B* and *GRIN2C* were searched. Information relating to the cSNPs was obtained from the SNP database (www.ncbi.nlm.nih.gov/SNP; dbSNP Build 130) of the National Center for Biotechnology Information (NCBI). cSNPs with unknown heterozygosity and minor allele frequencies (<5%) were excluded. Finally, 9 cSNPs were selected [*GRIN2A* (rs8049651, Leu425Leu; rs9806806, Tyr730Tyr); *GRIN2B* (rs7301328, Pro122Pro; rs35025065, Asp447Asp; rs1805522, Ile602Ile; rs1806201, Thr888Thr; rs1805247, His1399His); and *GRIN2C* (rs689730, Ala33Ala; rs3744215, Arg1209Ser)] for analysis. Genomic DNA was extracted from blood samples collected in EDTA using a commercially available Qiagen DNA extraction kit (Qiagen, Tokyo, Japan). Genomic DNA was amplified using primers for each cSNP (Table II). The PCR products were sequenced using an ABI PRISM 3730XL analyzer (PE Applied Biosystems, Foster City, CA, USA). Sequence data were analyzed using SeqManII software (DNASTAR Inc., Madison, WI, USA).

### Statistical analysis

The Hardy-Weinberg equilibrium (HWE) was assessed using SNPStats (http://bioinfo.iconcologia.net/snpstats) (12). Multiple logistic regression models (codominant, dominant and recessive) were used to calculate the odds ratio (OR), 95% confidence interval (CI) and corresponding P-values, with age and gender as covariables. To analyze the associations of cSNPs and haplotypes, SNPStats, HapAnalyzer and HelixTree (Golden Helix, Inc., Bozeman, MT, USA) were used.

## Results

Of the nine cSNPs of *GRIN2A*, *GRIN2B* and *GRIN2C* genes examined, eight were polymorphic and one (rs35025065) was monomorphic (Table III). The genotype distributions of the eight cSNPs were in HWE (P>0.05, data not shown).

As shown in Table III, there was no significant difference in the genotypic frequency of polymorphism rs8049651 between the DLS (CC 87%, CG 13% and GG 0%) and control groups (CC 87%, CG 11% and GG 1%; P=0.76). Additionally, the other seven cSNPs were not associated with DLS. Via the Gabriel method (13), two cSNPs (rs1805247 and 1806201 of GRIN2B) were used to construct a one linkage disequilibrium (LD) block (data not shown). Three haplotypes in the block had frequencies >0.1. However, there was no significant difference in haplotype frequencies between the DLS and control groups (data not shown).

However, when DLS patients were divided into two groups according to clinical characters based on Cobb’s angle (<20° or ≥20°) and lateral listhesis (<6 mm or ≥6 mm), associations were observed between the *GRIN2B* and *GRIN2C* polymorphisms and clinical characteristics. As shown in Table IV, rs689730 of *GRIN2C* was associated with Cobb’s angle in the codominant (P=0.038; OR, 2.72; 95% CI, 0.98–7.55) and dominant models (P=0.022; OR, 3.79; 95% CI, 1.15–12.54). The present study showed that the CT or TT genotype frequency with Cobb’s angle ≥20° was ∼2-fold higher than with Cobb’s angle <20°. In the analysis of lateral listhesis, rs7301328 of *GRIN2B* showed significant differences in the codominant (P=0.0027; OR, 3.21; 95% CI, 1.41–7.29), dominant (P=0.015; OR, 3.91; 95% CI, 1.22–12.51) and recessive models (P=0.015; OR, 5.39; 95%CI, 1.26–23.08). The results indicated that the CC genotype frequency with lateral listhesis ≥6 mm was ∼4-fold higher than with lateral listhesis <6 mm (Table IV).

## Discussion

A number of individuals present degenerative diseases of the spine, such as spondylolisthesis, lateral listhesis, spinal stenosis and degenerative scoliosis with increasing age (3,14). Scoliosis is a spinal deformity with lateral curvature or angulation and may be accompanied by rotation of the affected vertebrae in the vertical axis (15).

Complicated DLS induces severe back or leg pain with neurological deficits by compressing neural elements and causing nerve root ischemia (16). Despite uncertainty with regard to the etiology of DLS, degeneration of the spinal column occurs in the development of the disease (3). Considering that the etiology of DLS is unclear, the identification of candidate genes associated with the development of DLS may potentially be used to identify at-risk individuals. Therefore, the potential of *NMDAR2* genes as candidate genes in the development of DLS was investigated through a case-control analysis in the current study.

Glutamate is the most prominent excitatory neurotransmitter in the CNS. It has been studied intensively in a number of neuropathological conditions, including stroke, epilepsy and neurodegenerative diseases (17,18). Glutamate exerts its effects through ionotropic ligand-gated ion channels and metabotropic G-protein-coupled glutamate receptors. The ionotropic receptors are further divided into NMDA, α-amino-3-hydroxy-5-methylisoxazole-4-propionic acid (AMPA) and kainate receptors (19–21). Spencer *et al*(6) conducted a review of NMDA-type glutamate signalling.

In the current study, 9 cSNPs in the genes *GRIN2A*, *GRIN2B* and *GRIN2C* were analyzed to assess the association between these polymorphisms and DLS. The *GRIN2A*, *GRIN2B* and *GRIN2C* genes are located on chromosomes 16p13.2, 12p12 and 17q25, respectively. In the coding regions of each gene, 15, 22 and 14 cSNPs have been discovered, respectively. Of these 51 cSNPs, 9 exhibited heterozygosity >0.1 (www.ncbi.nlm.nih.gov/SNP, dbSNP Build 130) and were selected for analysis in thist study. Overall, none of the genotype frequencies or haplotype distributions of the NMDRs (*GRIN2A*, *GRIN2B* and *GRIN2C*) were observed to be associated with DLS.

Several studies have shown associations between SNPs of *GRIN2A* and/or *GRIN2B* and neurological or psychiatric diseases, such as Huntington’s disease (22), schizophrenia (23), bipolar disorder (24) and Alzheimer’s disease (25). In addition, Kim *et al*(26) reported that rs1806201 of *GRIN2B* was associated with alcoholism in a Korean population, but rs18054247 was not. However, Tadic *et al*(27) reported that rs1806201 was not associated with alcohol dependence, alcohol withdrawal-induced seizures and delirium tremens in a Caucasian population.

The possible associations between nine cSNPs of *GRIN2A*, *GRIN2B* and *GRIN2C* genes and DLS were investigated, but the results were negative. To compare each genotype frequency in various ethnic populations, the human SNP database (www.ncbi.nlm.nih.gov-SNP, dbSNP Build 130) was searched. The genotype distributions of all cSNPs in the control group were similar to those of the Asian populations, particularly the Japanese population (data not shown).

When the patients were divided into two groups according to clinical characteristics (Cobb’s angle and lateral listhesis), *GRIN2B* and *GRIN2C* were associated with the severity of DLS. As shown in Table IV, rs689730 of GRIN2C was associated with Cobb’s angle in the codominant (P=0.038) and dominant models (P=0.022). Furthermore, rs7301328 of *GRIN2B* was associated with lateral listhesis in the codominant (P=0.003), dominant (P=0.015) and recessive models (P=0.015).

The present results indicate that *GRIN2A*, *GRIN2B* and *GRIN2C* do not affect the development of DLS in the Korean population. However, significant associations were observed between *GRIN2C* and Cobb’s angle and between *GRIN2B* and lateral listhesis in DLS.

## Figures and Tables

**Table I.** Characteristics of DLS and control subjects.

Factor	DLS (n=70)	Controls (n=141)
Age (years, mean ± SD)	67.63±7.05	66.4±8.23
Gender (male/female)	10/60	25/116
Cobb’s angle (°, mean ± SD)	17.07±2.95	NA
Lateral listhesis (mm, mean ± SD)	4.78±2.30	NA

DLS, degenerative lumbar scoliosis; NA, not applicable.

**Table II.** Sequences of primers for cSNPs of *GRIN2A*, *GRIN2B* and *GRIN2C* genes.

Gene	SNP	Direction	Sequence (5′→3′)	Size (bp)
*GRIN2A*	rs8049651, Leu425Leu	Forward	GATTTGCCTCTCCAGAAATCAG	321
		Reverse	CTATTTCAAAGGGTTGGGCACG	
	rs9806806	Forward	TGCATGCATTTACCTCCTAACA	380
	Tyr730Tyr	Reverse	AATGGGAGCAGATAGGAACTGA	
*GRIN2B*	rs7301328, Pro122Pro	Forward	CGAGAAAGATGATTTCCACCAT	391
		Reverse	GATTAATTGCTGGCCTATCCAC	
	rs35025065, Asp447Asp	Forward	GGTCTGTTGCCTGCTTTATTTC	322
		Reverse	CAGGTTCCATTGATTTTCTTCC	
	rs1805522, Ile602Ile	Forward	TGAGAGTCCCTGGAAAAGAGAG	392
		Reverse	CCAGAAACCTGGTCCACATATT	
	rs1806201, Thr888Thr	Forward	TTGTGGTCATTTCTAGCCTCTC	371
		Reverse	CCGAACGTTCTCTCTACCTCAC	
	rs1805247, His1399His	Forward	CCTACGCCCACATGTTTGAGAT	381
		Reverse	GGTTTTTGTTGTTAGGCACACA	
*GRIN2C*	rs689730, Ala33Ala	Forward	AACCTCTGTCTCTCTCCCTGTG	394
		Reverse	GGAGATGAAGTCAAGGATCTGG	
	rs3744215, Arg1209Ser	Forward	CTGTCTGTCCTCACCTTCCACC	392
		Reverse	CACCATGATTTGAGGCTACTGA	

SNP, single nucleotide polymorphisms.

**Table III.** Genotype frequencies of cSNPs in *GRIN2A*, *GRIN2B* and *GRIN2C* genes.

			DLS		Control				
Gene	SNP	Genotype	n	Freq.	n	Freq.	Models	OR (95% CI)	P-value
*GRIN2A*	rs8049651, Leu425Leu	C/C	61	0.87	123	0.87	Codominant	0.88 (0.40–1.94)	0.76
		C/T	9	0.13	16	0.11	Dominant	0.99 (0.42–2.35)	0.99
		T/T	0	0.00	2	0.01	Recessive	0.00 (0.00–NA)	0.17
	rs9806806, Tyr730Tyr	C/C	63	0.90	120	0.85	Codominant	0.60 (0.26–1.37)	0.20
		C/G	7	0.10	18	0.13	Dominant	0.64 (0.25–1.59)	0.32
		G/G	0	0.00	3	0.02	Recessive	0.00 (0.00–NA)	0.11
*GRIN2B*	rs7301328, Pro122Pro	C/C	12	0.18	31	0.22	Codominant	0.86 (0.57–1.29)	0.46
		G/C	32	0.47	61	0.43	Dominant	0.91 (0.49–1.69)	0.77
		G/G	24	0.35	49	0.35	Recessive	0.69 (0.32–1.46)	0.32
	rs1805522, Ile602Ile	A/A	5	0.07	7	0.05	Codominant	0.93 (0.57–1.51)	0.76
		G/A	16	0.23	41	0.29	Dominant	0.84 (0.45–1.56)	0.58
		G/G	49	0.70	93	0.66	Recessive	1.22 (0.36–4.16)	0.75
	rs1806201, Thr888Thr	A/A	14	0.21	34	0.24	Codominant	0.89 (0.58–1.36)	0.60
		G/A	37	0.54	72	0.51	Dominant	0.95 (0.48–1.87)	0.88
		G/G	17	0.25	34	0.24	Recessive	0.77 (0.38–1.57)	0.47
	rs1805247, His1399His	A/A	46	0.66	91	0.65	Codominant	0.84 (0.50–1.40)	0.50
		A/G	23	0.33	42	0.30	Dominant	0.96 (0.53–1.77)	0.91
		G/G	1	0.01	8	0.06	Recessive	0.20 (0.02–1.68)	0.08
*GRIN2C*	rs689730, Ala33Ala	C/C	36	0.54	81	0.58	Codominant	1.28 (0.77–2.13)	0.34
		C/T	26	0.39	56	0.40	Dominant	1.14 (0.63–2.06)	0.66
		T/T	5	0.07	3	0.02	Recessive	3.31 (0.76–14.48)	0.10
	rs3744215, Arg1209Ser	G/G	26	0.39	44	0.31	Codominant	0.97 (0.64–1.47)	0.88
		G/T	27	0.40	74	0.52	Dominant	0.75 (0.41–1.39)	0.36
		T/T	14	0.21	23	0.16	Recessive	1.38 (0.66–2.91)	0.40

SNP, single nucleotide polymorphism; cSNPs, coding SNPs; DLS, degenerative lumbar scoliosis; freq, frequency; OR, odds ratio; 95% CI, 95% confidence interval; NA, not applicable.

**Table IV.** Genotype frequencies of rs689730 and rs7301328 in DLS patients with Cobb’s angle or lateral listhesis.

A, Cobb’s angle								
		<20°		≥20°				
SNP	Genotype	n	Freq.	n	Freq.	Model	OR (95% CI)	P-value
GRIN2C,	C/C	15	0.75	21	0.45	Codominant	2.72 (0.98–7.55)	0.038
rs689730,	C/T	4	0.20	22	0.47	Dominant	3.79 (1.15–12.54)	0.022
Ala33Ala	T/T	1	0.05	4	0.09	Recessive	1.82 (0.18–17.97)	0.590

B, Lateral listhesis								
		<6 mm		≥6 mm				
SNP	Genotype	n	Freq.	n	Freq.	Model	OR (95% CI)	P-value
GRIN2B,	C/C	3	0.08	9	0.32	Codominant	3.21 (1.41–7.29)	0.003
rs7301328,	G/C	18	0.45	14	0.50	Dominant	3.91 (1.22–12.51)	0.015
Pro122Pro	G/G	19	0.48	5	0.18	Recessive	5.39 (1.26–23.08)	0.015

DLS, degenerative lumbar scoliosis; SNP, single nucleotide polymorphism; freq, frequency. OR, odds ratio; 95% CI, 95% confidence interval.

## References

[1] Aebi M (2005). The adult scoliosis. Eur Spine J.

[2] Ploumis A, Transfledt EE, Denis F (2007). Degenerative lumbar scoliosis associated with spinal stenosis. Spine J.

[3] Oskouian RJ, Shaffrey CI (2006). Degenerative lumbar scoliosis. Neurosurg Clin N Am.

[4] Daffner SD, Vaccaro AR (2003). Adult degenerative lumbar scoliosis. Am J Orthop (Belle Mead NJ).

[5] Skerry TM (2008). The role of glutamate in the regulation of bone mass and architecture. J Musculoskelet Neuronal Interact.

[6] Spencer GJ, McGrath CJ, Genever PG (2007). Current perspectives on NMDA-type glutamate signalling in bone. Int J Biochem Cell Biol.

[7] Weinberg RJ (1999). Glutamate: an excitatory neurotransmitter in the mammalian CNS. Brain Res Bull.

[8] Merle B, Itzstein C, Delmas PD, Chenu C (2003). NMDA glutamate receptors are expressed by osteoclast precursors and involved in the regulation of osteoclastogenesis. J Cell Biochem.

[9] Hinoi E, Takarada T, Yoneda Y (2004). Glutamate signaling system in bone. J Pharmacol Sci.

[10] Mason DJ (2004). Glutamate signalling and its potential application to tissue engineering of bone. Eur Cell Mater.

[11] Lin TH, Yang RS, Tang CH, Wu MY, Fu WM (2008). Regulation of the maturation of osteoblasts and osteoclastogenesis by glutamate. Eur J Pharmacol.

[12] Solé X, Guinó E, Valls J, Iniesta R, Moreno V (2006). SNPStats: a web tool for the analysis of association studies. Bioinformatics.

[13] Gabriel SB, Schaffner SF, Nguyen H (2002). The structure of haplotype blocks in the human genome. Science.

[14] Liao JC, Chen WJ, Chen LH, Niu CC (2009). Outcome of the L5-S1 segment after posterior instrumented spinal surgery in degenerative lumbar diseases. Chang Gung Med J.

[15] Schlenk RP, Kowalski RJ, Benzel EC (2003). Biomechanics of spinal deformity. Neurosurg Focus.

[16] Arbit E, Pannullo S (2001). Lumbar stenosis: a clinical review. Clin Orthop Relat Res.

[17] Fisher M (1997). Characterizing the target of acute stroke therapy. Stroke.

[18] Moult PR (2009). Neuronal glutamate and GABAA receptor function in health and disease. Biochem Soc Trans.

[19] Tanabe Y, Masu M, Ishii T, Shigemoto R, Nakanishi S (1992). A family of metabotropic glutamate receptors. Neuron.

[20] Hollmann M, Heinemann S (1994). Cloned glutamate receptors. Annu Rev Neurosci.

[21] Yoneda Y, Kuramoto N, Kitayama T, Hinoi E (2001). Consolidation of transient ionotropic glutamate signals through nuclear transcription factors in the brain. Prog Neurobiol.

[22] Arning L, Saft C, Wieczorek S (2007). NR2A and NR2B receptor gene variations modify age at onset in Huntington disease in a sex-specific manner. Hum Genet.

[23] Tang J, Chen X, Xu X (2006). Significant linkage and association between a functional (GT)n polymorphism in promoter of the N-methyl-D-aspartate receptor subunit gene (GRIN2A) and schizophrenia. Neurosci Lett.

[24] Szczepankiewicz A, Skibinska M, Rybakowski J (2009). Lack of association of three GRIN2B polymorphisms with bipolar disorder. World J Biol Psychiatry.

[25] Seripa D, Matera MG, Franceschi M (2008). Association analysis of GRIN2B, encoding N-methyl-D-aspartate receptor 2B subunit, and Alzheimer’s disease. Dement Geriatr Cogn Disord.

[26] Kim JH, Park M, Yang SY (2006). Association study of polymorphisms in N-methyl-D-aspartate receptor 2B subunits (GRIN2B) gene with Korean alcoholism. Neurosci Res.

[27] Tadic A, Dahmen N, Szegedi A (2005). Polymorphisms in the NMDA subunit 2B are not associated with alcohol dependence and alcohol withdrawal-induced seizures and delirium tremens. Eur Arch Psychiatry Clin Neurosci.

